# Pooled prevalence and associated factors of overweight/obesity among under-five children in 46 low- and middle-income countries: A cross-sectional analysis of Demographic and Health Surveys data (2015–2024)

**DOI:** 10.1371/journal.pgph.0006575

**Published:** 2026-08-24

**Authors:** Dejen Kahsay Asgedom, Fentahun Bikale Kebede, Simachew Getaneh Endalamew, Bewuketu Terefe, Solomon Keflie Assefa, Etsay Woldu Anbesu

**Affiliations:** 1 Department of Public Health, College of Medicine and Health Science, Samara University, Samara, Ethiopia; 2 Strategic Affairs Executive Office, Ministry of Health, Addis Ababa, Ethiopia; 3 Department of Veterinary Epidemiology and Public Health, School of Veterinary Medicine, Bahir Dar University, Bahir Dar, Ethiopia; 4 College of Medicine and Health Science, University of Gondar, Gondar, Ethiopia; 5 School of Public Health, The University of Queensland, Brisbane, Australia; 6 Department of Public Health, Pawi Health Science College, Pawi, Ethiopia; Hamadan University of Medical Sciences School of Public Health, IRAN, ISLAMIC REPUBLIC OF

## Abstract

Overweight and obesity remain major global public health challenges, particularly among children under five years of age in low- and middle-income countries (LMICs). This study aimed to assess the pooled prevalence of overweight/obesity and its associated factors among children aged 6–59 months in 46 LMICs. We used the most recent nationally representative demographic and health surveys (2015–2024) from forty-six LMICs. A total of 393,638 weighted samples of children aged 6–59 months were included. Childhood overweight and obesity were defined as weight-for-height greater than two and three standard deviations above the WHO reference population median, respectively. Multilevel log-binomial regression models were employed. An adjusted risk ratio (RR) with a 95% confidence interval (CI) was used to select variables that had a significant association with childhood overweight/obesity. A p value < 0.05 was used to declare statistical significance. The data were analyzed with R software (version 4.5.1). The pooled prevalence of overweight/obesity was 3% (95% CI: 2.44, 3.58), with significant regional variation ranging from 1.62% in West Africa to 10% in the Europe and Central Asia (95% CI: 5.26, 17.84). Male sex (RR = 1.07, 95% CI: 1.03, 1.11), larger-than-average birth size (RR = 1.66, 95% CI: 1.54, 1.78), facility delivery (RR = 1.20, 95% CI: 1.13, 1.27), maternal smoking (RR = 1.61, 95% CI: 1.38, 1.87), maternal overweighed/obesity (RR = 2.37, 95% CI: 2.19-2.56), media exposure (RR = 1.05, 95% CI: 1.01, 1.10), and rich (RR = 1.10, 95% CI: 1.04, 1.16) household wealth were factors associated with increased risk. Children aged 24–59 months had lower risk compared to those aged 6–23 months. Protective factors included current breastfeeding (RR = 0.91, 95% CI: 0.87, 0.95), interpregnancy intervals 18–23 months (RR = 0.77, 95% CI: 0.71, 0.83), and ≥ 24 months (RR = 0.87 95% CI: 0.83, 0.91), and multiple births (RR = 0.67, 95% CI: 0.57, 0.79). Contextually, children in medium–human development index (HDI) (RR = 1.80, 95% CI: 1.62, 2.00) and higher–HDI countries (RR = 3.57, 95% CI: 3.00, 4.24) had elevated risk. Compared to West Africa, all other regions showed significantly higher risk, with Europe & Central Asia demonstrated the strongest association (RR = 3.37, 95% CI: 2.90, 3.91). The pooled prevalence of overweight/obesity among under-five children in LMICs is relatively low compared to high-income nations, yet substantial regional variation exists. Male sex, larger size at birth, maternal smoking, maternal overweight/obesity, higher household wealth quantiles and higher-HDI were associated with increased risk of childhood overweight/obesity, whereas current breastfeeding and longer interpregnancy intervals were protective. These findings highlight the importance of close monitoring of the problem and region-specific interventions targeting modifiable risk factors.

## Introduction

Overweight/obesity is a condition characterized by excessive fat deposits in the body [1]. According to the World Health Organization (WHO), a child is considered overweight if their weight-for-height is more than two standard deviations (SDs) above the median and obese if it is more than three SDs above the median [2]. Overweight/obesity remains a major global public health challenge, particularly among children under the age of five in low- and middle-income countries (LMICs) [3]. In 2024, an estimated 34 million children under five years of age were classified as overweight/obese [4]. Once viewed primarily as a concern for high-income countries, overweight/obesity is on the rise in LMICs. In Africa, the number of overweight/obese children has increased by 12.1% since 2000, and nearly half of all those under five years of age who were overweight or obese in 2024 resided in Asia [4].

The health consequences of overweight or obesity are well documented. Although overweight/obesity is often not recorded as a direct cause of death, it is estimated to contribute to more than 3.7 million child deaths globally [4,5]. In addition to mortality, childhood overweight/obesity is associated with immediate and long-term risks. It is associated with an earlier onset of noncommunicable diseases (NCDs), such as type 2 diabetes; adverse psychological outcomes, including stigma and quality of life; and impaired educational performance [6]. Furthermore, overweight/obesity in children strongly predicts their persistence into adulthood, perpetuating a cycle of elevated NCD risk. The economic ramifications are equally profound, with the projected global cost reaching US$3 trillion annually by 20230 and exceeding US$18 trillion by 20260 in the absence of effective interventions [7].

Overweight or obesity arises from an imbalance between energy intake from the diet and energy expenditure through physical exercise. The condition is typically multifactorial and driven by a complex interplay of environmental factors, such as limited access to affordable and healthy food and psychosocial and genetic factors [6]. The LMICs face a profound double burden of malnutrition (DBM). Children in these settings are particularly vulnerable and are often subjected to inadequate early nutrition while being increasingly exposed to energy-dense, nutrient-poor foods. This dietary pattern combined with insufficient physical activity drives a sharp increase in childhood overweight or obesity, all while the challenges of undernutrition remain unresolved [8,9].

The WHO has long urged action on the global obesity crisis, endorsing 2012 targets (extended to 2030) to halt childhood overweight and obesity increases aimed at the DBM. In 2022, the 75^th^ World Assembly adapted the WHO acceleration plan to stop obesity, fostering policy momentum, country implementation, and accountability. In December 2025, the WHO issued a guideline on glucose-like peptide-1 (GLP-1) therapies to integrate safe pharmaceutical options into comprehensive adult obesity care as part of lifelong chronic disease management [10,11]

Despite these efforts, the increasing prevalence of pediatric overweight or obesity cannot be overlooked if long-term nutritional goals are to be achieved effectively. There is an urgent need for more research and population-based surveys to generate up-to-date evidence on this underrecognized poorly understood form of malnutrition. In addition, understanding the factors influencing the prevalence of childhood overweight/obesity is crucial for improving their health and achieving the SDGs related to reducing by two-third premature mortality due to NCDs and ending any form of malnutrition by 2030 in LMICs [12]. Therefore, this study aimed to assess the pooled prevalence of overweight/obesity and its associated risk factors among under-five children in 46 selected LMICs. We analyzed secondary data from recent demographic and health surveys conducted in these countries, which provide comprehensive anthropometric, dietary, and sociodemographic data for both children under five years of age and their mothers. These findings are expected to offer valuable insights for researchers, health policy makers, and other stakeholders working to address this problem in low- and middle-income countries.

## Methods and materials

### Study settings, period, and data sources

This study assessed the prevalence of overweight/obesity among children aged 6–59 months across 46 low- and middle-income countries via the DHS conducted between 2015 and 2024. The DHS program established by USAID in 1984 has conducted over 350 surveys in more than 90 countries across eight phases. The DHS data received logistical and financial support from international development partners, including the WHO, the United Nations International Children’s Emergency Fund (UNICEF), and the United Nations Population Fund (UNFPA), with internal technical assistance provided by the ICF International and the national government. These surveys provide standardized indicators for comparing health outcomes and tracking progress toward global development goals (GDGs). The DHS program utilizes nationally representative, population-based surveys to collect data on key indicators such as fertility, child health, nutrition, and HIV/AIDS via uniform questionnaires and standardized protocols [13]. To ensure cross-national comparability, the program applied uniform questionnaires and standardized protocols, with all complete datasets made publicly available at https://www.dhsprogram.com/.

### Study design and sampling techniques

The DHS data are cross-sectional by design. Sample selection methods are detailed in the published DHS report, including sample size computations. These surveys utilized a multistage cluster sampling approach, stratifying urban and rural areas into strata that form the foundation for selecting primary sampling units (PSUs), i.e., clusters of households. A fixed number of households per PSU were then chosen for interviews, targeting men aged 15–59 years, women aged 15–49 years, and children aged under five years. A detailed explanation of the sampling process is available in the DHS-8 manual [13].

### Population and sample size

In this study, all children aged 6–59 months across the 46 LMICs were considered the source population, whereas all children aged 6–59 months within the selected enumeration area composed the study population. Data were extracted from the kids record (KR) file. After the exclusion of children whose BMI z scores were missing and/or those flagged cases (z scores ≤ -5 SD and ≥ 5 SD) [14], a final weighted sample of 393,638 children was included in the study (S1 Table).

### Study variables

#### Dependent variable.

The dependent variable for this study was childhood overweight/obesity. According to the WHO cutoff, a child was classified as overweight/obese if their weight-for-height z–score (WHZ) exceeded + 2 standard deviations (SDs), otherwise not overweight/obese. Any WHZ z–scores beyond the plausible values (≤ -5 SD and ≥ 5 SD) were considered flagged cases and were removed from the analysis [2,14–16].

#### Independent variables.

On the basis of previous studies [15,17,18] and availability in the DHS datasets, we considered the independent variables as follows: **maternal and household factors** include mothers’ age (15–24,25–34, 35–49) years**,** education (no education, primary, secondary, higher)**,** current marital status (married, unmarried)**,** maternal nutritional status (underweight, normal, overweight/obese), smoking history (no, yes), wealth index (poor, middle, rich), mass media exposure (no, yes)**, and** family size (1–4, ≥ 5); **obstetric and infant characteristics** include mother’s age at first pregnancy (< 19, ≥ 19), parity (primipara, multipara), number of ANC visits (zero, at least one), place of delivery (home, at health facility), sex of a child (male, female), twin (singleton, multiple), birth order (1^st^, 2^nd^, ≥ 3), preceding birth interval in months (<18, 18–23, ≥ 24), perceived size of the child at birth (smaller than average, average, larger than average), current breastfeeding (no, yes), and community-level factors.

### Operational definitions

The predictor **media exposure** is a composite variable of frequency of listening radio, watching television and reading newspaper, in which households are said to have media exposure if they have been exposed to either listening radio or watching television or reading newspaper for at least one week and have not been combined to avoid exposure to all of the above media sources [19].

**The variable wealth index** was categorized as follows: the original categories “poorest” and “poorer” were combined into “poor”; “middle” remained unchanged; and “richest” and “richer” were combined into “rich” [19].

The variable **maternal educational level** was categorized as follows: the original categories “no education, incomplete primary, complete primary, incomplete secondary, complete secondary, and higher” were recoded to “no education, primary, secondary/ higher.”

The variable **infant size at birth** was categorized as follows: the original categories “very small” and “smaller than average” were combined into “smaller than average”; “average” remained unchanged; and “very large” and “larger than average” were combined into “larger than average.”

**Maternal nutritional status** was defined as underweight if BMI < 18.5 kg/m^2^, normal between 18.5 kg/m2 and 24.99 kg/m^2^, and overweight/obese if > 25 kg/m^2^ and above 30 kg/m^2^.

The variable **Human Development Index (HDI)** value is a composite statistic used to rank countries into four tiers of human development on the basis of long and healthy life (life expectancy), education (mean years of schooling and expected years of schooling), and standard of living (gross national income per capita). The HDI is used to categorize countries into four socioeconomic groups: very high, high, medium, and low HDIs [20]. Since there was no country in the very high HDI category, we categorized it into high, medium, and low HDIs.

**Country income level:** The country income variable was determined on the basis of the World Bank’s classification, which categorizes countries into low-income, lower-middle-income, and upper-middle-income groups [21]. A country is considered low income if its gross national income (GNI) per person was $1,135 or less in 2022. Countries with a GNI per capita falling between $1,136 and $4,465 are classified as having lower-middle income. The GNI per capita for upper- middle-income countries ranges from $4,466 to $13,845 [22].

**Geographic subregions:** The countries were classified according to the WHO, DHS and UNICEF [23,24] into the following subregions: West Africa, East Africa, South & Central Africa, East Asia & Pacific, Europe & Central Asia, South Asia, Latin America & Caribbean.

### Data management and analysis

The data were analyzed via R software version 4.5.1. To account for the unequal contribution of children from different countries, a sample weighting was performed for each nation before further analysis. The background characteristics of the sample population are presented as weighted frequencies and percentages. The pooled prevalence of overweight/obesity among children under five years of age in LMICs was estimated via the meta-analysis function metaprop() from the meta R package [25] and was presented via a forest plot. Prior to model fitting, chi-square bivariable tests were performed to assess the associations between child overweight/obesity and the predictors. Independent variables found to be associated with childhood overweight/obesity at a p value < 0.25 in the bivariable analysis were included in the multivariable models. Before fitting the final models, we assessed multicollinearity among covariates via the variance inflation factor (VIF) [26]. The results indicated no concern of collinearity, with a maximum variance inflation factor (VIF) of 2.83 for the human development index (HDI) variable and a mean variance inflation factor (VIF) of 1.5 across all covariates. To account for temporal heterogeneity across the pooled surveys, survey year was included as fixed effect in the multilevel models.

Owing to the inherent hierarchical nature of the DHS data, whereby children are nested within enumeration areas (EAs), the assumption of independent observation was violated. Thus, children living in the same EAs had a similar likelihood of experiencing the outcome of interest as those living in other EAs within the same country. To account for this clustering effect and the resulting correlation of outcomes within EAs, a multilevel mixed-effects modeling approach was employed [27]. Therefore, to identify risk factors associated with childhood overweight/obesity, a multilevel mixed-effects log–binomial regression model was applied.

We then fitted four hierarchical logistic models: the null model/model I (without independent variables), Model II (only individual-level variables (sociodemographic variables), Model III (only community-level variables), and Model IV (both individual-level and community-level variables). Since the models were nested, the likelihood ratio (LR) test was used for model comparison. Hence, the model with the lowest likelihood ratio (LR) values was considered the best fitted model.

In this context, the need for a multilevel mixed-effects modeling approach was assessed via the intraclass correlation (ICC).


$$\begin{array}{c} ICC=\frac{\sigma^{2}\mu }{\left(\sigma^{2}\mu +\pi^{2}/3\right)} \end{array}$$

where $\sigma^{2}\mu$ is the variance of the random parameter at the cluster level, which represents the amount of unobserved heterogeneity between clusters, and $\frac{\pi^{2}}{3}$ represents the within cluster variability. An ICC value greater than 0.05 justified the fit of a multilevel mixed-effects model [28,29].

The **proportional change in variance (PCV)** was used to quantify the proportion of total variance in the outcome explained by the final model.,


$$\begin{array}{c} 𝐏𝐂𝐕=\;\frac{\left({𝐕}_{0}-\;{\;\sigma }_{x}^{2}\right)*100}{{𝐕}_{0}} \end{array}$$

where V_0_ is the variance of the null model and where $\sigma_{x}^{2}\;$ is the variance of each model at each level with variables.

The unobserved heterogeneity across was measured via the **median risk ratio (MRR).**


$$\begin{array}{c} MRR=𝐞𝐱𝐩\left(\sqrt{2{\;\sigma }_{x}^{2}}\;\Phi^{-1}\left(0.75\right)\right)\; \end{array}$$

where ${\;\sigma }_{x}^{2}$ is the variance of each model and where $\Phi^{-1}$ is the inverse of the standard normal cumulative distribution function. Finally, findings are presented as risk ratios (RRs) with 95% confidence intervals (CIs). The threshold for statistical significance in all multivariable analyses was set to a p value < 0.05.

### Ethical consideration

The analysis was based on secondary, deidentified data obtained from the DHS Program. To ensure ethical compliance, the authors adhered to the DHS Program protocol: all data were handled without access to personally identifiable information, which was removed prior to public release by the DHS Program. Therefore, ethical approval and participant consent were waived. Formal authorization for data use was secured from the DHS Program’s institutional review board prior to data download from https://www.dhsprogram.com.

## Results

### Background characteristics of the study participants

The mean maternal age was approximately 29 years (DS ± 6), with 215,028 (55%) of the study participants being between the ages of 25 and 35 years. With respect to educational attainment, the largest proportion 197, 583 (51%) of mothers had completed secondary education and above. The majority of mothers, 369,174 (94%), were married. In terms of nutritional status, nearly one-third 121,137(31%) were overweight/obese. The smoking prevalence was low, with 99% of mothers reporting no tobacco use. Half of the mothers,193,313 (49%), had mass media exposure, whereas nearly half 177,951 (45%) mothers were classified as poor (Table 1).

**Table 1:** Sociodemographic characteristics of the study participants at LMICs.

Variable	Frequency	Percent
**Mother’s age (years)**		
15-24	110,506	28
25-34	215,028	55
35-49	68,104	17
**Mother’s education**		
No education	108,137	27
Primary	87,917	22
Secondary	155,654	40
Higher	41,929	11
**Current marital status**		
Married	369,174	94
Unmarried	24,464	6
**Mother’s nutritional status**		
Underweight	52,713	13
Normal	219,787	56
Overweight/obese	121,137	31
**Smoking status**		
No	390,265	99
Yes	3,373	1
**Mass media exposure**		
No	200, 326	51
Yes	193,313	49
**Wealth index**		
Poor	177,951	45
Middle	78,185	20
Rich	137,501	35
**Family size**		
≤4	98,936	25
>4	294,702	75

### Obstetric and children characteristics

Among the 393, 638 children included in the study, 201,791 (51%) were males. Ninety-eight percent of the births were singleton, and more than half, 210,347 (53%), of the infants were born after an interpregnancy interval (IPI) of 24 months or longer. The majority of the children, 218,260 (63%), had an average size at birth. The majority, 314,404 (80%), of the mothers were multiparous, and 126,152 (32%) had their first birth before the age of 19 years. Sixty-nine percent of the mothers attended antenatal care, and 282,840 (72%) delivered at health facilities (Table 2).

**Table 2:** Obstetrics and child-related characteristics in LMICs.

Variable	Frequency	Percent
**Parity**		
Primipara	79,233	20
Multipara	314,404	80
**Age at first birth (years)**		
<19	126,152	32
≥19	267,486	68
**ANC visit during pregnancy**		
No	154,998	39.38
At least one	238,640	60.62
**Place of delivery**		
At home	110,797	28.15
At health facility	282,840	71.85
**Child’s sex**		
Male	201,791	51
Female	191,847	49
**Child’s age**		
<2 years	134,032	34
2-3 years	85,946	22
≥3 years	173,660	44
**Twin status**		
Single birth	384,893	98
Multiple birth	8,745	2
**Birth order**		
1st	121,214	31
2nd	106,924	27
≥3	165,501	42
**Birth interval (months)**		
<18	145,435	37
18-23	37,856	10
≥24	210,347	53
**Birth size**		
Small	45,742	13
Average	218,260	63
Large	81,475	24
**Currently breastfeeding**		
No	174,545	44
Yes	219,093	56

### Contextual factors

Most of the respondents, 290,384 (74%), resided in lower-middle income countries, and 285,842 (73%) were from medium-HDI nations. Geographically, half 197,473 (50%) of the participants were from South Asia, and the majority of the participants, 273,068 (69%), resided in rural areas (Table 3).

**Table 3:** Contextual characteristics of the participants in LMICs.

Variable	Frequency	Percent
**Income classification**		
Low	81,112	21
Lower-middle	290,384	74
Upper-middle	22,142	6
**Human Development Index**		
Low	91,273	23
Medium	285,842	73
High	16,523	4
**Geographic region**		
West Africa	68,010	17
East Africa	60,617	15
South/Central Africa	31,871	8
East Asia/Pacific	11,653	3
Europe/Central Asia	9,509	2
South Asia	197,473	50
Latin America/Caribbean	14,505	4
**Residence**		
Urban	120,570	31
Rural	273,068	69
**Total (N)**	**393,638**	**100**

### Prevalence of childhood overweight/obesity in LMIC

**Fig 1:**
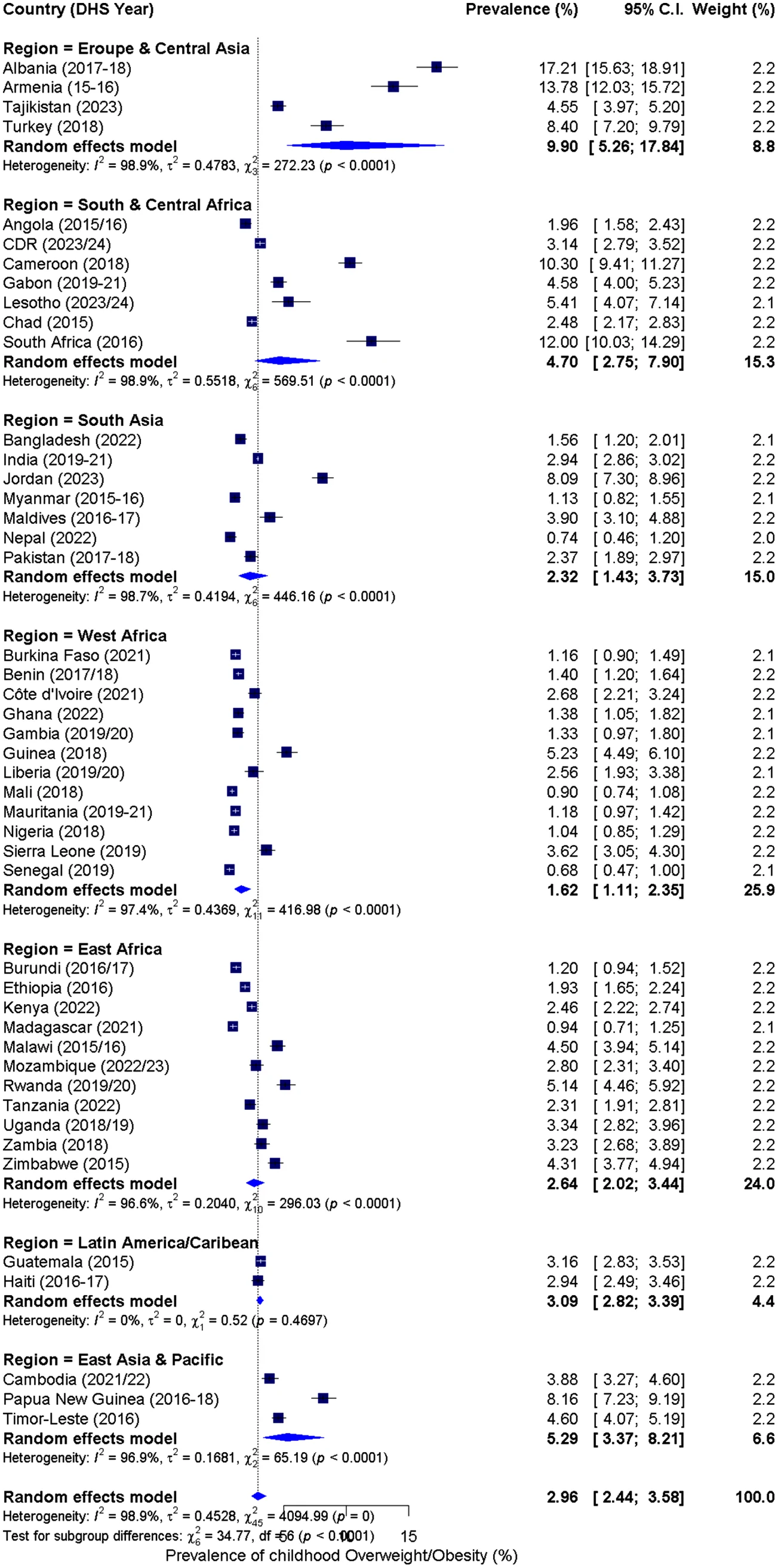
Pooled estimate of childhood overweight/obesity in LMICs (2015-2024).

The pooled prevalence of overweight or obesity among children aged 6–59 months in the LMICs was 2.96% (95% CI: 2.44, 3.58), with significant regional variations (heterogeneity I^2^ = 98.9%). The Europe and Central Asia region had the highest pooled prevalence, at 9.90% (95% CI: 5.26, 17.84). The West Africa region presented the lowest pooled prevalence of overweight/obesity, at 1.62% (95% CI: 1.11, 2.35). At the country level, Albania had the highest prevalence of childhood overweight/obesity (17.21%), followed by Armenia (13.78%) and South Africa (12%), whereas Senegal had the lowest prevalence (0.68%) (Fig 1).

The following subgroup analysis examines prevalence by country development level (human development index (HDI) and income group), which complement the geographic findings above. The prevalence of overweight/obesity among children aged 6–59 months in high-HDI countries was 8.72% (95% CI: 5.79–12.91) (S1 Fig). The prevalence of childhood overweight or obesity among children aged 6–59 months in upper-middle income countries was 7.84% (95% CI: 4.78–12.03), followed by lower-middle income countries (3.23%; 95% CI: 2.47–4.22) and low-income countries (3.10%; 95% CI: 2.37–4.04) (S2 Fig).

### Factors associated with childhood overweight/obesity in LMICs

To identify the factors associated with overweight/obesity, generalized linear mixed-effects models with binomial family and log-link functions were used. A model with individual-level and community-level factors (Model IV) was found to be the best-fit model since it had the smallest log-likelihood ratio (LLR = -49, 313.263) (Table 4). Therefore, factors associated with childhood overweight/obesity were identified on the basis of the best fit model (Table 4). Variables such as child sex, child age, birth size, child twin, interpregnancy interval (IPI), current breast feeding, place of delivery, maternal smoking history, maternal nutritional status, media exposure, wealth index, country’s income level, HDI, and subregion were significantly associated with overweight/obesity among children under five years of age.

**Table 4:** Sociodemographic factors associated with overweight/obesity among children aged 6–59 months at the LMIC.

Variable	Null model	Model I	Model II	Model III
		RR (95% CI)	RR (95% CI)	RR (95% CI)
**Child sex**				
Female		1		1
Male		1.07(1.03, 1.12) *		1.07 (1.03-1.11) *
**Child age in months**				
6-23		1		1
24-34		0.54 (0.50, 0.57) *		0.67 (0.64-0.71) *
≥35		0.62 (0.55, 0.66) *		0.63 (0.60-0.66) *
**Birth size**				
Smaller than average		1		1
Average		1.31 (1.22, 1.41) *		1.32 (1.24-1.42) *
Large than average		1.50 (1.38, 1.62) *		1.66 (1.54-1.78) *
**Birth interval in months**				
<18		1		1
18-23		0.96 (0.86, 1.07)		0.77 (0.71-0.83) *
≥24		1.12 (1.02, 1.22) *		0.87 (0.83-0.91) *
**Child twin**				
Single		1		1
Multiple		0.54 (0.41, 0.69) *		0.67 (0.57-0.79) *
**ANC visit**				
No		1		1
At least one		0.85 (0.79, 0.91) *		1.04 (0.99-1.09)
**Place of delivery**				
At home a		1		1
At health facility		1.13 (1.06, 1.20) *		1.20 (1.13-1.27) *
**Currently breastfeeding**				
No		1		1
Yes		1.06 (1.01, 1.13) *		0.91 (0.87-0.95) *
**Maternal age**				
15-24		1		1
25-34		0.88 (0.82, 0.93) *		0.98 (0.94-1.03)
35-49		0.91 (0.84, 0.98) *		1.04 (0.97-1.12)
**Maternal education**				
No education		1		1
Primary		1.26 (1.18, 1.34) *		0.99 (0.93-1.05)
Secondary/Higher		1.41 (1.33, 1.50) *		1.04 (0.98-1.10)
**Mother nutritional status**				
Underweight		1		1
Normal		1.80 (1.66, 1.96) *		1.65 (1.54-1.77) *
Overweight/Obese		2.40 (2.05, 2.45) *		2.37 (2.19-2.56) *
**Maternal smoking**				
No		1		1
Yes		1.73 (1.45, 2.05) *		1.61 (1.38-1.87) *
**Media exposure**				
No		1		1
Yes		1.06 (1.01, 1.11) *		1.05 (1.01-1.10) *
**Wealth index**				
Poor		1		1
Middle		0.99 (0.93, 1.05)		1.05 (0.99-1.11)
Rich		0.96 (0.91, 1.01)		1.10 (1.04-1.16) *
**Residence**				
Urban			1	1
Rural			0.82 (0.78-0.85) *	0.98 (0.93-1.03)
**Country’s income group**				
low			1	1
Lower-middle			0.65 (0.58-0.72) *	0.64 (0.57-0.71) *
Upper-middle			0.49 (0.41-0.57) *	0.49 (0.41-0.59) *
**HDI**				
Low			1	1
Medium			1.74 (1.58-1.93) *	1.80 (1.62-2.00) *
High			4.14 (3.56-4.82) *	3.57 (3.00-4.24) *
**Subregion**				
West Africa			1	1
East Africa			1.47 (1.34-1.60) *	1.53 (1.37-1.70) *
South & Central Africa			2.16 (1.97-2.36) *	2.37 (2.13-2.63) *
East Asia & Pacific			2.75 (2.44-3.10) *	2.50 (2.19-2.86) *
Europe &Central Asia			3.39 (3.00-3.84) *	3.37 (2.90-3.91) *
South & South East			1.82 (1.67-1.99) *	2.00 (1.81-2.21) *
Latin America & Caribbean			1.75 (1.50-2.03) *	1.55 (1.31-1.84) *
**Survey year**				
2015-2018			1	1
2019-2022			0.69 (0.65-0.74) *	0.66 (0.62-0.71) *
2023-2024			0.73 (0.68-0.79) *	0.63 (0.57-0.69) *

Compared with female children, male children had a 7% greater risk of being overweight/obese (AOR = 1.07, 95% CI: 1.03– 1.11). Compared with children aged 6–23 months, children aged 24–35 and 36–59 years had 33% (RR = 0.67, 95% CI: 0.64–0.71) and 35% (RR = 0.63 95% CI: 0.60–0.66) lower risks of overweight/obesity, respectively. Having a larger-than-average size at birth was associated with a 51% higher likelihood of overweight/obesity (RR = 1.66, 95% CI: 1.54–,1.78), whereas having an average size at birth was associated with a 32% higher risk (RR = 1.32, 95% CI: 1.24–,1.42) than having smaller size at birth. Compared with those born after a short interval of less than 18 months, children born after an interval of 18–23 years had a 23% lower likelihood of being overweight/obese (RR = 0.77, 95% CI: 0.71– 0.83), whereas those born after 24 months or more had a 13% lower likelihood of being overweight/obese (RR = 0.87, 95% CI: 0.83–0.91). Compared with home delivery, delivery at health facilities was associated with a 14% higher risk of childhood overweight/obesity (RR = 1.14, 95% CI: 1.08 –1.21). Being currently breastfed was associated with a 9% lower likelihood of being overweight/obese (RR = 0.91, 95% CI: 0.87–0.95). Twins or multiples had a 33% lower risk of overweight/obesity than singleton children did (RR = 0.67, 95% CI: 0.57-0.79). Born at health facility was associated with 20% higher likelihood of overweight/obesity (RR = 1.20, 95% CI: 1.13-1.27).

Compared with their counterparts, children whose mothers had a history of smoking were 61% more likely to be overweight/obese (RR = 1.61, 95% CI: 1.38,1.87). Compared with children whose mothers were underweight, children of overweight/obese mothers had a 2.3 times higher risk of overweight/obese themselves (RR = 2.37, 95% CI: 2.19-2.56), whereas children from mother of normal BMI mothers were 36% more likely to be overweight/obese (RR = 1.65, 95% CI: 1.54,1.77).

Media exposure was associated with a 5% higher risk of childhood overweight/obesity (RR = 1.05, 95% CI: 1.01-1.10). Compared with those from poor households, children from rich households were 10% more likely to be overweight/obese (RR = 1.10, 95% CI: 1.04-1.16).,

Living in countries with a medium human development index (HDI) was associated with an 80% higher likelihood of overweight/obesity (RR = 1.80, 95% CI: 1.62-2.00), whereas living in countries with a high HDI was associated with approximately 4–fold higher risk of overweight/obese (RR = 3.57, 95% CI: 3.00-4.24) than residing in countries with a low HDI. Compared with those in low-income countries, children in lower-middle income countries had a 36% lower risk of overweight/obesity (RR = 0.64, 95% CI: 0.57-0.71), whereas those in lower-upper income countries had a 51% lower risk (RR = 0.49 95% CI:0.41-0.59).

Compared with children in West Africa, children in East Africa had a 53% higher risk of overweight or obesity (RR = 1.53, 95% CI: 1.37-1.70). Similarly, a significantly greater risk was observed in other regions, including children in South & Central Africa (RR = 2.37, 95% CI: 2.13-2.63), East Asia & Pacific (RR = 2.50, 95% CI: (2.19-2.86), Europe & Central Asia (RR = 3.37, 95% CI: 2.90-3.91), South Asia (RR = 2.00, 95% CI: 1.81-2.21), and Latin America/Caribbean (RR = 1.55, 95% CI: 1.31-1.84) (Table 4).

#### Random effect analysis.

The random effect estimates were determined by fitting four models (the null model (Model I), Model II, Model III, and Model IV)). The null model revealed significant variability in the risk of overweight/obesity among children under five years in LMICs ($\sigma^{\text{2}}=\text{0}.\text{8}\text{7})$. The intraclass correlation coefficient (ICC) for the null model was 21%, meaning that 21% of the total variation in individuals under five degrees of overweight/obesity was attributed to community-level differences, whereas 83% of the variability in childhood overweight/obesity was explained by individual differences. As indicated by the MRR = 2.43 in the null model, children residing in clusters with a high risk of overweight/obesity were 2.43 times more likely to have an outcome than those living in low-risk communities were. The variance explained by individual-level factors was 85.06%, that explained by community-level factors was only 26.44%, and that explained by both community- and individual–level factors was 87.36% (Table 5).

**Table 5:** Random effect analysis and model comparison.

Parameters	null model (model I)	Model II	Model III	Model IV
Cluster level variance ($\sigma^{2}$)	0.87	0.13	0.64	0.11
ICC%	20.9%	3.80%	16.280%	3.24%
PCV%		85.06%	26.44%	87.36%
MRR	2.43	1.41	2.14	1.37
LLR	-72,374.205	- 50,215.427	-66087.383	-49,313.263

## Discussion

This study aimed to assess the pooled prevalence and risk factors for overweight and obesity among children aged 6–59 months in the LMIC. The results revealed that the pooled prevalence of overweight/obesity was 2.96% (95% CI: 2.44, 3.58), whereas Europe and Central Asia presented the highest combined prevalence of overweight/obesity at 10% (95% CI: 5.26, 17.84). Historically, research on malnutrition has focused mainly on undernutrition. However, there is increasing concern about the double burden of malnutrition in LMICs [3,14]. Although childhood obesity is viewed as a public health problem that primarily affects high-income nations [30], perhaps because undernutrition is less prevalent there, recent evidence has suggested that children in disadvantaged environments also face a significant risk of becoming overweight/obese [31]. Given the growing evidence linking poverty and childhood overweight/obesity, it is critical for low- and middle-income nations to prioritize this issue in their nutritional policies. In line with past reports, the present findings showed that the pooled prevalence of overweight/obese LMICs is relatively low [3,14,32,33]. However, to fully grasp the scale of the problem, further analysis using longitudinal data is recommended.

This study revealed that variables such as the child’s sex, age, size at birth, twin status, interpregnancy interval, current breastfeeding, place of delivery, maternal smoking history, maternal nutritional status, media exposure, wealth quantiles, country’s income level, HDI, and subregion were significantly associated with overweight/obesity among children aged 6–59 months. Being male was associated with a 7% higher risk of childhood overweight/obesity. This finding is in line with the results reported from studies in Ethiopia [34], Cameroon [35], Uganda [36], and France [18]. This association may be attributed to a combination of factors, including biological differences (as boys tend to have a large body size). Females typically have higher concentrations of circulating leptin, a hormone that suppresses appetite and promotes energy expenditure [37,38]. In contrast, males tend to have higher androgen levels, with a suppressive effect on leptin production [39]. However, our findings are inconsistent with those of a multinational study comprising 12 nations [40]. Therefore, to understand the association between sex and childhood overweight/obesity, further studies are needed.

In line with past reports [17,34,36], this study revealed that an older child’s age was associated with a lower likelihood of overweight/obesity. This might be due to increased physical activity, with more exposure to open playgrounds, potentially lowering their chance of being overweight/obese [17]. Additionally, the transaction from infancy (characterized by faster fat accumulation) to early child hood (characterized by lean mass growth) may naturally reduce the risk of overweight/obesity in this age group [41]. Furthermore, factors such as continued breastfeeding and dietary diversity might also contribute to this protective effect [17].

The present study revealed that, compared with singleton children, twins and multiples had a 33% lower risk of overweight/obesity. This protective effect most likely stems from biological factors associated with multiple pregnancies, including restricted intrauterine growth (IUGR) (leading to lower birth weight) compared with that associated with singleton pregnancies.

We observed that a large size at birth was significantly associated with a greater risk of subsequent overweight/obesity among children aged 6–59 months, which is consistent with studies in SSA [17,34], England [42], Brazil [43], China [44], and many other epidemiological studies [40,45,46]. The biological plausibility of this association is supported by evidence linking overweight to later-life adiposity, including large waist and hip circumferences [47]. Macrosomia often results from excessive in utero nutrition, leading to fetal alterations that predispose individuals to postnatal overweight/obesity [48]. In addition, the mechanism of the association between birth weight/macrosomia and childhood overweight/obesity is that a greater maternal nutrient supply to the fetus elevates insulin levels, leading to increased adiposity and long-term developmental changes in fetal adipose tissue and long-term effects during childhood [49]. Therefore, pediatricians should prioritize nutritional counseling growth tracking for large newborns, enabling timely interventions such as activity encouragement. Public health strategies targeting maternal diet during pregnancy could curb population-level trends [44].

In line with previous research findings [15,50,51], this study revealed that breast feeding was associated with 9% lower risk of childhood overweight/obesity. There are many possible explanations for these results. First, breast milk provides balanced calories and nutrients, including sugars, water, proteins, and fats, tailored to infant needs [52]. Second, its composition adapts over time and with the maternal diet, unlike formula, which often exceeds the requirements for protein and fats [53,54]. Moreover, excess early protein and fat from formula promote adiposity [54]. Furthermore, breast milk unequally contains bioactive compounds such as leptin ghrelin, which regulate adipocyte proliferation and differentiation [55,56]. Overall, these factors make breast milk superior for preventing excess fat accumulation.

Regular disposable income enables junk food purchases, shifting infant feeding toward obesogenic patterns despite better knowledge [57]. In LMIC such as SSA [17] and South Asia [15], China [58], Denmark [59], and Norway [60], revealed that high-SES children face 20–50% elevated overweight odds from such exposures, which is consistent with our 19% finding. This implies context-specific interventions, i.e., nutritional literacy for all mothers plus regulating the marketing of unhealthy foods to higher SES groups [57].

The present study revealed a protective effect of longer interpregnancy intervals (IPIs) against childhood overweight/obesity. Children born after 18–23 months had a 23% lower likelihood of being overweight/obese, and those born at 24 months or longer presented a 14% lower risk than those with short IPIs (<18 months). This finding was in line with the findings reported in previous studies [61] and the WHO recommendations of waiting at least 24 months after a live birth before the next pregnancy to reduce the risk of adverse maternal, perinatal, and infant outcomes [62]. This might be explained by the fact that moderate to long interpregnancy interventions allow for maternal physiological recovery. This recovery includes normalization of BMI, replenishment of nutritional stores, and completion of breast feeding. These factors may reduce the likelihood of fetal metabolic programming that favors excess adiposity and helps mitigate perinatal risks, including macrosomia, a known precursor for childhood obesity [40,63].

The present study revealed a significant positive association between maternal smoking history and childhood overweight/obesity, with affected children facing a 61% higher likelihood of overweight/obesity. This finding aligns with evidence from previous studies [64,65], where perinatal and postnatal maternal smoking has consistently been linked to increased adiposity in offspring, often through mechanisms such as fetal programming, altered appetite regulation, and nicotine-induced metabolic changes [64,65]. Therefore, screening for maternal smoking history during pediatric assessment could enable targeted counseling on nutrition and physical activity. Further studies should explore mediating pathways, such as epigenetic modifications, to refine preventive strategies.

In line with past research findings [15,66,67], the current study revealed a significant positive association between maternal BMI and that of offspring. This association might be explained by the intricate interplay of physiological, environmental, and behavioral factors [66,67]. This intergenerational correlation in nutritional status holds important interest for nutritional policy and planning efforts.

In this study, we found that children in households from higher wealth quantiles presented an elevated risk of overweight/obesity. This positive association between SES and childhood obesity aligns with prior findings from South Asia [15] and Northern Indian cohorts [68], Denmark [59], Norway [60], and SSA [17,36]. This might be attributed to enhanced financial gains being associated with obesogenic behaviors, such as increased caloric intake, diets high in meats and dairy, and reduced physical activity [69]. Furthermore, regular disposable income enables junk food purchases, shifting infant feeding toward obesogenic patterns despite better knowledge [57]. In LMIC such as SSA [17] and South Asia [15], China [58], Denmark [59], and Norway [60], revealed that high-SES children face 20–50% elevated overweight odds from such exposures, which is consistent with our 10% finding. This implies context-specific interventions, i.e., nutritional literacy for all mothers plus regulating the marketing of unhealthy foods to higher SES groups [57]. However, this pattern contrasts with other findings, where low household income was associated with a higher risk of childhood overweight/obesity [69]. Consequently, interventions targeting childhood overweight/obesity must account for parental socioeconomic circumstances to deliver tailored dietary and lifestyle recommendations.

This study demonstrated a robust positive association between national human development index (HDI) levels and childhood overweight/obesity risk, with children in medium-HDI countries having an 80% higher risk and those in high-HDI countries having a 4-fold more risk. This aligns with global epidemiological trends; high-HDI (HDI ≥ 0.8) nations have a 9.5% prevalence of child obesity vs. 7.6% in low-HDI settings [70]. Similarly, the transition from low to high HDI is correlated with a 7.45% increase in obesity, which is linked to factors such as urbanization and dairy/milk consumption [71]. Finally, this study revealed that, compared with children in West Africa, children living in Europe & Central Asia, East Asia & Pacific, South & Central Africa, Latin America & Caribbean, South Asia, and East Africa presented approximately three-, 2.5-, two-three-, two-, two-, and two-fold higher risks of overweight/obesity, respectively. These findings were consistent with findings from previous studies in the region [3,17,72] and WHO reports [33]. This discrepancy could be attributed to variation in socioeconomic and sociocultural factors, including differences in cultural feeding practices and food preferences for children under five as well as methodological approaches and time period differences across studies.

The study successfully shifts the paradigm of public health in developing countries, proving that the Double Burden of Malnutrition (DBM) is actively present. It emphasizes that interventions in LMICs can no longer solely focus on wasting and stunting. Public health strategies must be dual-purpose: promoting physiological recovery via optimal birth spacing (≥24 months) and protective breastfeeding, while strictly regulating junk food marketing targeted at affluent socio-economic groups in medium-to-high HDI environments where pediatric obesity is actively concentrating.

### Strengths and limitations of this study

To the best of our knowledge, this is the largest study focusing on childhood overweight in LMICs using population-level data. While previous DHS-based studies emphasized the significance of the double burden of malnutrition [3], this analysis offers a broad, comparative perspective of the problem and robustly identifies various individual-level and community-level variables. These insights are valuable for guiding further studies and enhancing ongoing intervention strategies. Another key strength of this study is its application of a multilevel mixed-effects modeling approach to control cluster-level dependency, thereby preventing misleading inferences and ensuring the valid interpretation of the results. Moreover, this study utilized the most recent nationally representative datasets, which strengthens the external validity of the results.

It is important to acknowledge several key limitations of this study. First, the data were collected at different time points across the 46 countries, which may affect the comparability of the overweight/obesity prevalence rates. Second, because the underlying DHS data are cross-sectional, the study is entirely incapable of establishing true causality. It can only identify correlations and therefore, residual confounding may explain some of the observed associations. Third, while the study covers macro-level contextual and sociodemographic metrics, the DHS does not collect direct, standardized metrics on actual daily caloric intake, physical activity levels, or the precise consumption of energy-dense ultra-processed foods, which are the fundamental biological drivers of the double burden of malnutrition. Further studies incorporating dietary recalls, accelerometry, and biomarker data are needed to complement our findings.

## Conclusion

The pooled prevalence of overweight/obesity among children aged 6–59 months in LMICs was relatively low compared with that reported in high-income nations and in previous studies conducted in the region. The prevalence varied significantly across the seven subregions, ranging from 1.62% in West Africa to 10% in Europe and Central Asia. Male sex, younger age, large size at birth, maternal smoking, maternal nutritional status, rich wealth quantiles, living in medium- and high-HDI countries, living in Europe & Central Asia, East Asia & Pacific, South & Central Africa, Latin America & Caribbean, South Asia, and East Africa were associated with increased childhood overweight/obesity, whereas current breastfeeding and longer IPIs were correlated with a lower risk of overweight/obesity among children under the age of five. These findings highlight the importance of close monitoring of the problem and the identified risk factors.

**Recommendations and policy implications**

- To prevent childhood overweight/obesity in LMICs, policy makers should prioritize promoting optimal birth spacing.
- To prevent the epidemic in LMICs, policy makers and health stakeholders should pay special attention to modifiable risk factors such as breastfeeding and maternal nutritional status, and maternal smoking.
- We also advise that region-specific strategies be developed for infants in Europe & Central Asia, East Asia & Pacific, South & Central Africa, Latin America & Caribbean, South Asia, and East Africa, and upper-middle-income and high-HDI countries.
- Targeted initiatives aimed at addressing socioeconomic disparities, such as wealth inequality, can also play a pivotal role in preventing childhood overweight/obesity in LMICs.
- Policy maker should design HDI level–based interventions, since children from higher HDI level country had consistently higher risk of overweight/obesity.
- Health policy maker should adapt community–based nutrition programs for higher–income communities where prevalence was concentrated.

## Supporting information

S1 Table Sample size for the prevalence of overweight/obesity and associated factors among under five children in LMICs, 2015–2024. (DOCX)

S1 Fig Forest plot for the prevalence and of overweight/obesity among under five children in LMICs by country’s HDI value, 2015–2024. (DOCX)

S2 Fig Forest plot for the prevalence of overweight/obesity among under five children in LMICs by country’s income group, 2015–2024. (DOCX)

## Abbreviations

CIs: confidence intervals
DHS: Demographic and Health Survey
HDI: human development index
ICC: intraclass correlation
IPIs: interpregnancy interval
LMIC: low- and middle-income countries
RR: risk ratio.
