# Supplementary material for: Pooled prevalence and associated factors of overweight/obesity among under-five children in 46 low- and middle-income countries: A cross-sectional analysis of Demographic and Health Surveys data (2015–2024)
Source: PLOS Glob Public Health. 2026 Aug 24;6(8):e0006575. doi: 10.1371/journal.pgph.0006575 (PMC13502597; doi:10.1371/journal.pgph.0006575)
Supplement: Supplementary file 1 [file pgph.0006575.s001.docx]

**S1 Table. Sample size for pooled prevalence and associated factors of overweight/obesity among under five children in LMIC, 2015-2024.**

| **Country (DHS Year)** | **Weighted sample** | **Unweighted sample** |
| --- | --- | --- |
| Albania (2017-18) | 2,028 | 2,233 |
| Armenia (15-16) | 1,343 | 1,395 |
| Angola (2015/16) | 4,137 | 4,359 |
| Bangladesh (2022) | 3,659 | 3,666 |
| Burkina Faso (2021) | 5,099 | 5,131 |
| Benin (2017/18) | 10,691 | 10,659 |
| Burundi (2016/17) | 5,587 | 5,426 |
| CDR (2023/24) | 8,675 | 9,544 |
| Côte d'Ivoire (2021) | 3,809 | 4,223 |
| Cameroon (2018) | 4,144 | 3,956 |
| Ethiopia (2016) | 8,467 | 7,903 |
| Gabon (2019-21) | 4,456 | 4,735 |
| Ghana (2022) | 3,613 | 3,910 |
| Gambia (2019/20) | 3,018 | 3,265 |
| Guinea (2018) | 2,942 | 3,016 |
| Guatemala (2015) | 9,808 | 9,733 |
| Haiti (2016-17) | 4,696 | 4,924 |
| India (2019-21) | 178,958 | 182,344 |
| Jordan (2023) | 4,189 | 4,557 |
| Kenya (2022) | 13,642 | 15,418 |
| Cambodia (2021/22) | 3,297 | 3,445 |
| Liberia (2019/20) | 1,876 | 2,156 |
| Lesotho (2023/24) | 851 | 954 |
| Madagascar (2021) | 5,097 | 5,137 |
| Mali (2018) | 12,513 | 12,473 |
| Myanmar (2015-16) | 3,360 | 3,786 |
| Mauritania (2019-21) | 8,757 | 8,679 |
| Maldives (2016-17) | 1,848 | 2,169 |
| Malawi (2015/16) | 4,601 | 4,791 |
| Mozambique (2022/23) | 3,566 | 8,417 |
| Nigeria (2018) | 8,339 | 2,339 |
| Nepal (2022) | 2,282 | 3,017 |
| Papua New Guinea (2016-18) | 3,028 | 3,705 |
| Pakistan (2017-18) | 3,077 | 3,435 |
| Rwanda (2019/20) | 3,523 | 3,576 |
| Sierra Leone (2019) | 3,505 | 3,980 |
| Senegal (2019) | 3,805 | 8,723 |
| Chad (2015) | 8,710 | 4,258 |
| Tajikistan (2023) | 4,378 | 5,257 |
| Timor-Leste (2016) | 5,329 | 1,899 |
| Turkey (2018) | 1,761 | 4,272 |
| Tanzania (2022) | 4,323 | 3,939 |
| Uganda (2018/19) | 3,888 | 927 |
| South Africa (2016) | 900 | 3,399 |
| Zambia (2018) | 3,310 | 4,368 |
| **Total** | **393,638** | **403,009** |
