# Supplementary material for: Pooled prevalence and associated factors of overweight/obesity among under-five children in 46 low- and middle-income countries: A cross-sectional analysis of Demographic and Health Surveys data (2015–2024)
Source: PLOS Glob Public Health. 2026 Aug 24;6(8):e0006575. doi: 10.1371/journal.pgph.0006575 (PMC13502597; doi:10.1371/journal.pgph.0006575)
Supplement: Supplementary file 3 [file pgph.0006575.s003.docx]

**S2 Fig. Forest plot for the pooled prevalence of childhood overweight/obesity among under five children in LMICs by income group, 2015-2024.**
